# LDMP-RENet: Reducing intra-class differences for metal surface defect few-shot semantic segmentation

**DOI:** 10.1371/journal.pone.0318553

**Published:** 2025-03-17

**Authors:** Jiyan Zhang, Hanze Ding, Zhangkai Wu, Ming Peng, Yanfang Liu

**Affiliations:** 1 College of Mathematics and Information Engineering, Longyan University, Longyan, China; 2 School of Computer Science, University of Technology Sydney, New South Wales, Australia; Najran University College of Computer Science andInformation Systems, SAUDI ARABIA

## Abstract

Given their fast generalization capability for unseen classes and segmentation ability at pixel scale, models based on few-shot segmentation perform well in solving data insuﬃciency problems during metal defect detection and in delineating refined objects under industrial scenarios. Extant researches fail to consider the inherent *intra-class differences* in data about metal surface defects, so that the models can hardly learn enough information from the support set for guiding the segmentation of query set. Specifically, it can be categorized into two types: the *semantic intra-class difference* induced by internal factors in metal samples and the *distortion intra-class difference* caused by external factors of surroundings. To address these differences, we introduce a **L**ocal **D**escriptor-based **M**ulti-**P**rototype **R**easoning and **E**xcitation **Net**work (**LDMP-RENet**) to learn the two-view guidance, i.e., the local information from the graph space and the global information from the feature space, and fuse them to segment precisely. Given the contribution of relational structure of graph space-embedded local features to the *Semantic Difference* obviation, a multi-prototype reasoning module is utilized to extract local descriptors-based prototypes and to assess relevance between local-view features in support-query set pairs. Meanwhile, since global information helps obviate *Distortion Difference* in observations, a multi-prototype excitation module is employed for capturing global-view relevance in the above pairs. Lastly, an information fusion module is employed to integrate the learned prototypes in both global and local views, thereby creating pixel-level masks. Thorough experiments are conducted on defect datasets, revealing the superiority of proposed network to extant benchmarks, which sets a new state-of-the-art.

## Introduction

Few-Shot Segmentation (FSS) models have potential applicability for detecting defects on metal surfaces. These models are capable of discerning unseen concepts from limited annotated examples [1,2]. And they effectively mitigate the data insuﬃciency issue, which arises from the pricey pixel-level annotations and scarcity of defects in the actual industrial settings. Beyond their rapid generalization capabilities, FSS models, enhanced by semantic segmentation modules [3–5], can accurately capture the location and structure of defects. Owing to their intensive prediction capability, these models are beneficial for industrial applications. This capability overcomes issues with vague location information from classification-based approaches [6–9]. It also addresses the boundary inflexibility resulting from object detection approaches, where regular bounding boxes are unable to accommodate defects of varying shapes (e.g. scratches and patches) [10,11].

**Fig 1:**
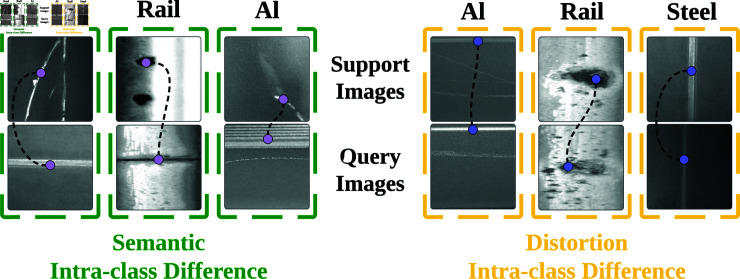
Inherent in metal surface defect data, we identify two primary types of intra-class differences that significantly affect metal surface defect detection. Firstly, the semantic intra-class difference is delineated through the examination of 3 support-and-query pairs, showcasing the distinct differences within the same defect categories. These differences are often due to diverse manufacturing processes, lighting conditions, or noise interference, leading to distinct appearances of defects like Steel, Rail, or Aluminum (Al). Secondly, the distortion intra-class difference is highlighted in another set of 3 support-and-query pairs, where defects exhibit differences caused by optical lens distortions or the specific perspective of the image capture. This can result in the altered shape, scale, and orientation of defect instances, further complicating the detection and classification process.

**Fig 2:**
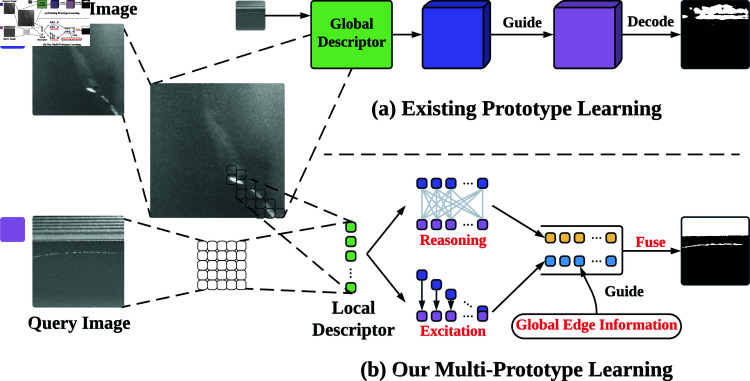
Comparison of traditional approaches and our multi-protorype learning. Firstly, traditional models extract features from image based prototypes. In contrast, LDMP-RENet employs local descriptor-based multi-prototype to represent more implicit local relations. Secondly, our model generates features in local (by Reasoning operation) and global views (by Excitation operation and Global Edge Infomation operation). The two differences are addressed separately after acquiring the local-view graph space features (represented by yellow squares) and the global-view features (represented by blue squares).

Aside from the single metal surface defect segmentation tasks [12], FSS-based models demonstrate their potential in generic surface defect segmentation tasks [13]. However, they neglect the inherent intra-class differences of metal defect samples, leading to inadequacy of the guidance knowledge learned from support set for segmenting the query set samples. We specifically categorize the intra-class differences [14] under two types inherent in metal defect data: The first type is semantic intra-class differences caused by intrinsic physical traits, where defects vary in fine-grained classes despite sharing the same metal origin. The second type is distortion intra-class differences, which are induced by external factors, mostly by perspective distortion. For a more intuitive understanding of the two differences, refer to Fig 1. To address this, we propose a **L**ocal **D**escriptor-based **M**ulti-**P**rototype **R**easoning and **E**xcitation **Net**work (**LDMP-RENet**), which consists of a Multi-Prototype Reasoning (MPR) module, a Multi-Prototype Excitation (MPE) module, and an Information Fusion Module (IFM) to produce suﬃcient guidance from multi-prototype-based support-and-query pairs to generate precise query masks. Unlike traditional approaches, deep local descriptors are utilized to embed features, replacing the image-level features with local descriptor-level ones [15] to enhance the manipulation flexibility (Fig 2 presents more detailed comparison).

For the semantic intra-class difference reduction, it is necessary to strengthen the perception of local-view information within identical classes [16]. The capability of Graph Convolutional Network (GCN) to handle structural correlations [17] facilitates the comprehensive analysis of semantic information embedded in local features. Motivated by this, we designed the MPR module to model the semantic correlations between support-query prototypes within a graph space generated by local descriptors. The relevance of consistent defect features in support-query pairs can be achieved through reasoning among graph nodes.

Next, the distortion intra-class differences are resolved using the MPE module. In such scenarios, differences can be effectively mitigated by comprehensive global-view features, as distortion-induced perceptual blurring still contains the semantic information distinguished intuitively. For example, the ability to recognize a person from afar simply by observing their eyes demonstrates that critical global semantic details remain identifiable. Enlightened by this, relevant defect features are directly activated in the feature space and global-view features are extracted by handling global edge information (GEI).

Lastly, the local and global features from the aforementioned two modules are integrated in the IFM. In this way, the guidance from the graph and feature spaces is enriched, which improves the defect segmentation accuracy. A more comparison and intuitive explanation of the LDMP-RENet framework can be found in Fig 2.

Our contributions can be summarized as follows:

1) We define two intra-class differences in FSS based metal defect tasks. Namely, semantic intra-class differences arise from the internal property within metal data, and distortion intra-class differences arise from external factors in data collection.
2) We propose the MPR and MPE modules to generate multi-prototype based guidance information and employ IFM to do pixel-level segmentation. Experimental results show that our features fused by graph and feature space can alleviate the two differences effectively.
3) Numerous experiments have shown that the mIoU and FB-IoU performances of LDMP-RENet exceed those of the popular metal surface defect and amateur FSS networks, reaching state-of-the-art levels.

## Related work

### Metal surface defect segmentation

Segmentation of metal surface defects is an important quality control task in the industrial production and manufacturing phases. Its goal is to categorize every pixel on metal surface images under designated semantic classes. The latest progress of metal semantic segmentation improves defect feature extraction by adopting multi-scale attention feature fusion modules [18]. Researchers have proposed a semantic prior and extremely eﬃcient dilated convolution network to detect metal surfaces pixel by pixel. The network attempts to solve the aforementioned problems through object detection combined with semantic segmentation for tackling various atypical defects [19]. DRNet [20] indicates that attention and weak supervision technologies are being leveraged to overcome the challenges of data scarcity and the high costs of manual labeling in metal surface defect detection models. By integrating the CG-FSDS paradigm, MFANet [21] addresses the problem that fine-grained segmentation methods make the dataset construction process arduous and time-consuming. Res12_SMGA [22] explores a multi-domain industrial few-shot defect recognition method based on attention embedding and fine-grained feature enhancement. Nonetheless, these methods rely overly on the information within classes, resulting in suboptimal adaptability in cases where intra-class differences are salient, which is usually because the metal surfaces have heterogeneous textural features.

### Few-shot segmentation

As an extended version of few-shot learning, FSS makes pixel-by-pixel prediction of unseen classes under sample size limitation. QPENet [23] focuses on the specific requirements of a query set and integrates query features into the generation process of foreground and background prototypes, resulting in customized prototypes suitable for specific queries. CSCANet [24] designs an few-shot semantic segmentation model based on the attention mechanism for remote sensing images characterized by complex backgrounds and tiny foreground objects. GLQSCA [25] focuses on the global-local information of the support image, aggregating segmentation labels from the support mask values (weighted by their similarity to all foreground prototypes (global information)) and the support pixels (local information). SiGR [26] mines latent contextual structures in query images to mitigate large appearance variations among objects from the same category. FIB [27] tackles the feature undermining issue of target class by exploiting information bottleneck theory to the few-shot semantic segmentation. Recent years have seen the introduction of graph-based FSS algorithm [13] for segmenting metal surface defects, which enables valid reasoning of the potential inter-defect correlations in the support-query pairs.

### Prototype-based learning

As a learning method based on metrics [28], the prototype-based learning intends to learn prototypes for representation. Through computation of the inter-prototype distance, the desired downstream tasks are then implemented. Employing a multi-prototype strategy, TPSN [29] addresses the inability of an individual prototype to precisely describe a class. To resolve the diﬃculties with classic prototype learning, a local descriptor-based multi-prototype learning is proposed [30]. Conventional few-shot multi-prototype learning methods for metal surface defect detection obtain predictions by analyzing image-level multi-prototypes in support-query pairs [31]. By contrast, our network employs local descriptors-based multi-prototype, mining the semantic relations of local descriptors to segmentation.

## Preliminary

### Problem definition

We train our model on the $D_{train}$ dataset and test it on the $D_{test}$ dataset, where the metal class sets $C_{train}$ and $C_{test}$do not intersect.

Different from conventional semantic segmentation approaches [32,33], FSS attempts to segment the objects in query set *Q* merely by using a few annotated samples in support set *S* and simulate few-shot settings by episodically training the model. To be specific, every episode encompasses each one *Q* and *S* sets with *K*-shot samples, denoted as:

1) a support set $S={\left\{\left(I_{s}^{k},M_{s,c}^{k}\right)\right\}}_{c\in C_{episode}}^{k=1,\ldots ,K}$. Here, $I_{s}^{k}$ stands for the *k*-th support image and $M_{s,c}^{k}$ signifies the *k*-th mask for category *c*. Furthermore, $C_{episode}$ refers to the category aligning with relevant episode.
2) a query set $Q=\left\{\left(I_{q},M_{q,c}\right)\right\}$, where $I_{q}$ stands for query image and $M_{q,c}$ refers to ground-truth mask for category *c*, which is known in the training process and unknown during testing.

Support-query pair contains merely a single defect category (one-way), while the rest defect classes are deemed as background.

Extension to the *K*-shot  ( *K* > 1 )  scenario, *K* support images with their labeled masks *S* and the query set *Q* are given. Through feature averaging, LDMP-RENet can be readily and rapidly extended to the new scenario. Afterwards, the masked $F_{s}$ is derived by:


$$\begin{array}{c} F_{s}=\frac{\overset{K}{\underset{k=1}{\sum }}\varphi (I_{s}^{k})\times M_{s,c}^{k}}{K}, \end{array}$$ (1)

with *φ* ( ⋅ )  standing for feature extraction. In this way, information from multiple shots are integrated and subsequent operations are simplified.

### Local descriptor-based multi-prototype

Classic manual descriptors have been growingly replaced by deep local descriptors [15], particularly in the field of few-shot classification. However, they are scarcely applied in FSS approaches. In this paper, an innovative local descriptors-based FSS strategy is put forward. Initially, the feature map $F\in {\mathbb{R}}^{c\times h\times w}$ is denoted as a local descriptor subset $X=\left\{x_{\left(i,j\right)}\right\}$, where  ( *i* , *j* )  , with  ( *i* , *j* )  representing a specific location in the map, and *i* ∈ ( 1 , . . . , *h* ) , *j* ∈ ( 1 , . . . , *w* ) , $x_{\left(i,j\right)}\in {\mathbb{R}}^{c}$. Besides, *c* , *h* and *w* refer separately to channel, height and width.

### Graph convolution network

Our network integrates graph convolution with the multi-prototype utilizing local descriptors, setting it apart from existing networks. Here, the graph structure is defined as *G* = ( *V* , *E* ) , where *V* denotes nodes and *E* edges. We proceed by defining the adjacency matrix *A* along with the degree matrix *D* , and thus we can formulate graph convolution as shown below:


$$\begin{array}{c} H^{\left(l+1\right)}=\sigma ({\tilde{D}}^{-\frac{1}{2}}Ã{\tilde{D}}^{-\frac{1}{2}}H^{\left(l\right)}\Theta^{\left(l\right)}), \end{array}$$ (2)

where $H^{\left(l+1\right)}$, $H^{\left(l\right)}$ represent the features of  ( *l* + 1 ) -th and *l*-th layers, respectively, *σ* signifies trainable parameters corresponding to $H^{\left(l\right)}$, stands for nonlinear activation function, ${\tilde{D}}_{ii}=\sum_{j}{Ã}_{ij}$.

Subsequently, the graph Laplacian matrix is incorporated to simplify the representation:


$$\begin{array}{c} \tilde{L}={\tilde{D}}^{-\frac{1}{2}}Ã{\tilde{D}}^{-\frac{1}{2}}. \end{array}$$ (3)

Therefore, can be expressed as:


$$\begin{array}{c} H^{\left(l+1\right)}=\sigma (\tilde{L}H^{\left(l\right)}\Theta^{\left(l\right)}). \end{array}$$ (4)

By employing the above steps, the inter-node correlations within graph structure are deduced, facilitating eﬃcient extraction of information.

## Method

**Fig 3:**
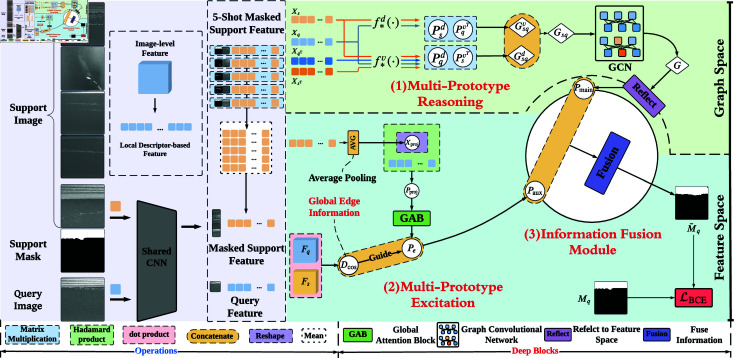
LDMP-RENet for 5-shot segmentation. (1) represents the process of Multi-Prototype Reasoning. (2) denotes the Multi-Prototype Excitation. Given $P_{main}$ and $P_{aux}$ from the above steps, we will get the prediction ${\tilde{M}}_{q}$ by (3) Information Fusion Module. Finally, we utilize BCE loss to train our model.

As shown in Fig 3, our **L**ocal **D**escriptor-based **M**ulti-**P**rototype **R**easoning and **E**xcitation **Net**work includes three modules, i.e., multi-prototype reasoning (MPR), multi-prototype excitation (MPE), and information fusion module (IFM). Our goal lies in utilizing the multi-prototype based on local descriptors for tackling the intra-class differences observed in metal surface defects.

Then, MPR is employed whose task is to produce local-view information $P_{main}$ of query image with the utilization of annotated support instances, to resolve semantic intra-class difference. Meanwhile, MPE is introduced to generate global-view information $P_{aux}$ which aims to mitigate the distortion intra-class difference. Finally, the local and global information are passed to IFM for fusion into a definitive guidance information, followed by mask ${\tilde{M}}_{q}$ prediction for query image.

Then, the segmentation loss $L_{BCE}$ is calculated by the binary cross entropy loss of predicted mask ${\tilde{M}}_{q}$ and the ground-truth $M_{q}$.


$$\begin{array}{c} L_{BCE}=\frac{1}{hw}\overset{h}{\underset{i=1}{\sum }}\overset{w}{\underset{j=1}{\sum }}BCE\left({\tilde{M}}_{q}(i,j),M_{q}(i,j)\right). \end{array}$$ (5)

### Multi-prototype reasoning

Despite great efforts to refine the prototype [34], the huge semantic intra-class difference remains inevitable due to the scarcity of support data and the diversity of metal surface defect appearances. Therefore, MPR is designed to process the local descriptors of support-query pair to multi-prototype through graph reasoning, aiming to mine semantic relations between defect appearances. The graph reasoning is more effective to mitigate the semantic intra-class difference, since it has the capability to capture the relations between distant regions of local descriptors. Moreover, we employ the local descriptors of transposed input features to equip the multi-prototypes with adaptability before graph reasoning. Such an adaptability enables the model to preemptively accommodate the diversity of defect appearances, which guides the network to extract more semantic features.

Given the query feature and support feature $F_{q},F_{s}\in {\mathbb{R}}^{c\times h\times w}$, we represent them using the corresponding local descriptors $X_{q},X_{s}\in {\mathbb{R}}^{c\times (hw)}$. Considering the query feature and support feature $F_{q},F_{s}\in {\mathbb{R}}^{c\times h\times w}$, we represent them using the corresponding local descriptors $X_{q},X_{s}\in {\mathbb{R}}^{c\times (hw)}$. Similarly, for the transposed query and support features $F_{q}^{\text{T}},F_{s}^{\text{T}}\in {\mathbb{R}}^{c\times w\times h}$, we derive the corresponding local descriptors $X_{q^{\text{T}}},X_{s^{\text{T}}}\in {\mathbb{R}}^{c\times (wh)}$. As shown in section “Local descriptor-based multi-prototype”, to embed the local descriptor into the graph space, we establish multiple-prototype $P_{s}^{v},P_{q}^{v}\in {\mathbb{R}}^{v\times (hw)}$ at the node level, and $P_{s}^{d},P_{q}^{d}\in {\mathbb{R}}^{d\times (hw)}$ at the channel level [13], with *v* and *d* signifying the node and channel quantities of multi-prototype, i.e., we embed multi-prototype from feature space into graph space along the above two directions. It is vital to note that the operations for inputs are analogous:


$$P_{q}^{v}=\phi \left(C\left(f_{v}\left(X_{q}\right)⊙f_{v}\left(X_{q^{\text{T}}}\right),f_{v}\left(X_{q}\right)\right)\right),$$ (6)

$$P_{q}^{d}=f_{d}\left(X_{q}\right),$$ (7)

where $f_{v}(\cdot )$ and $f_{d}(\cdot )$ denote 1-D convolution, respectively embedding the local descriptors to node level and channel level, *ϕ* ( ⋅ )  represents 1-D convolution reshaping the input channels from 2*d* to *d* dimensions, *C* ( ⋅ )  denotes concatenation and  ⊙  represents Hadamard product. Diverging from conventional graph reasoning methods, we have developed an adaptive process for obtaining $P_{s}^{v}$ and $P_{q}^{v}$, inspired by the Gram matrices based adjustment process [35]. Such an operation mines the potential semantic relation of the diversity of defect appearances, which guides model to link the semantic clues and result in mitigating the semantic intra-class difference.

Subsequently, we utilize the aforementioned four prototypes to produce the node-level relations $G_{sq}^{v}\in {\mathbb{R}}^{d\times v}$ and the channel-level relations $G_{sq}^{d}\in {\mathbb{R}}^{d\times v}$ of support-query pairs:


$$G_{sq}^{v}=P_{s}^{d}⊗P_{q}^{v^{\text{T}}},$$ (8)

$$G_{sq}^{d}=P_{q}^{d}⊗P_{s}^{v^{\text{T}}},$$ (9)

where  ⊗  represents matrix multiplication. After that, $G_{sq}^{v}$ and $G_{sq}^{d}$ are concatenated for generating relation $G_{sq}\in {\mathbb{R}}^{d\times 2v}$ of support-query pair:


$$\begin{array}{c} G_{sq}=C\left(G_{sq}^{v},G_{sq}^{d}\right). \end{array}$$ (10)

Following conversion of local descriptors to graph space, graph convolution is performed by to update graph nodes [13]. In both directions of $G_{sq}$, we employ the 1-D convolution for effective replacement of matrix multiplication ($\tilde{L}$ and $\Theta^{\left(l\right)}$) as outlined in .

Subsequently, the node-level 1-D convolution is utilized for fusion of 2*v* nodes from $G_{sq}$ into $G\in {\mathbb{R}}^{d\times v}$. In the context of *G*, the graph space is reprojected into the feature space using $P_{q}^{v}$:


$$\begin{array}{c} P_{main}=X_{q}⊕\tau \left(R(G⊗P_{q}^{v}\right)), \end{array}$$ (11)

where  ⊕  stands for element-wise addition, *τ* ( ⋅ )  represents 2-D convolution and normalization, *R* ( ⋅ )  reshapes the input to same size as $X_{q}$ and $P_{main}\in {\mathbb{R}}^{c\times (hw)}$ is the final output of MPR, encapsulating the semantic features of defects and addressing semantic intra-class differences.

### Multi-prototype excitation

A major diﬃculty with few-shot semantic segmentation is the intra-class differences, as displayed in Fig 1. Existing approaches attempt to tackle this diﬃculty by thoroughly mining the correlation of the foreground prototype with query image, that is, by excavating image-level prototype. Some methods employ additional background prototypes, which are though only capable of handling some highly related support-query pairs. For example, objects in the support and query images in Fig 1 (1st–3rd columns) share resembling local features, although their fine-grained classes vary. However, in Fig 1 (4th–6th columns), the presence of perspective distortion leads to loss of a few local features, and the model can hardly achieve query image segmentation based on the insuﬃcient support sample.

To address this, a MPE is employed to restore the potential defect features caused by distortion intra-class difference to generate an auxiliary multi-prototype in feature space. MPE contains two components: global attention block (GAB) and global edge information (GEI). GAB takes the foreground multi-prototype obtained from the local descriptors of support-query pair as input, and generates an activated multi-prototype. Using features at support and query image levels as its input, GEI generates cosine similarity to collect effective information from activated multi-prototype. Specifically, given the local descriptor-based support feature ***X_s_***, GAB obtains the foreground information $X_{proj}\in {\mathbb{R}}^{c\times 1}$ through mask averaging pooling [36]:


$$\begin{array}{c} X_{proj}={AVG}_{1D}\left(X_{s}\right)\text{, } \end{array}$$ (12)

where ${AVG}_{1D}(\cdot )$ represents 1-D average pooling with an output size of 1. where ${AVG}_{1D}(\cdot )$ represents 1-D average pooling (output size  = 1). Subsequently, the foreground information $X_{proj}$ is embedded into query descriptors to generate foreground multi-prototype $P_{proj}\in {\mathbb{R}}^{c\times (hw)}$:


$$\begin{array}{c} P_{proj}=R\left(X_{proj}\right)⊙X_{q}, \end{array}$$ (13)

where *R* ( ⋅ )  reshapes the input to same size as $X_{q}$.

**Fig 4:**
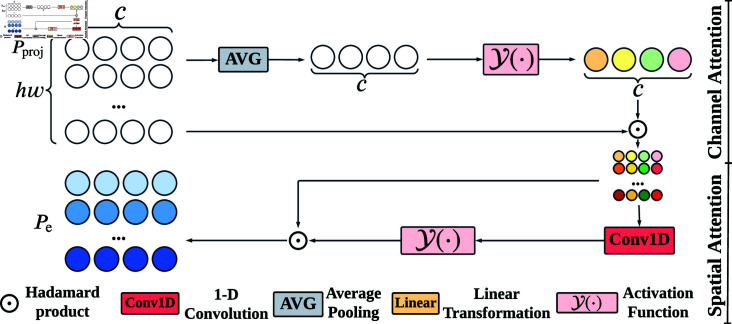
The overall structure of GAB. It activates foreground multi-prototype $P_{proj}$ via the channel and spatial attention and then yields the activated multi-prototype $P_{e}$.

Different from convolutional block attention module [37], GAB removes the max pooling in the channel attention module and reduces the number of nonlinear activation functions. The reason is that max pooling enhances the salience of the most prominent defective features, while an excess of nonlinear activation functions exacerbates the disparity with latent features, leading to the distortion intra-class difference. Thus, GAB (Fig 4) performs channel attention (without max pooling) and spatial attention on the foreground prototype separately to generate an activated multi-prototype $P_{c}\in {\mathbb{R}}^{c\times (hw)}$:


$$\begin{array}{c} P_{c}=P_{proj}⊙Y({AVG}_{2D}(P_{proj})), \end{array}$$ (14)

where $P_{c}\in {\mathbb{R}}^{c\times (hw)}$ indicates the features after channel attention, *Y* ( ⋅ )  stands for nonlinear activation function and reshaping, and ${AVG}_{2D}(\cdot )$ signifies 2-D average pooling.

After the channel attention, we proceed to spatial attention $P_{e}\in {\mathbb{R}}^{c\times (hw)}$:


$$\begin{array}{c} P_{e}=P_{c}⊙Y(f_{spatial}(P_{c})), \end{array}$$ (15)

where $f_{spatial}(\cdot )$ means 1-D convolution, and $P_{e}$ represents the activated information for metal defect samples, aiming to pinpoint regions of ambiguous perception. However, it is inevitable that background texture features will also be activated, potentially obscuring the clarity of the foreground information. Therefore, we employ the global edge information through calculating the cosine similarity $D_{cos}\in {\mathbb{R}}^{1\times h\times w}$ from $F_{s}$ and $F_{q}$, to collect effective information from defect area of $P_{aux}\in {\mathbb{R}}^{\left(c+1)\times (hw\right)}$:


$$D_{\text{cos}}=\frac{F_{s}\cdot F_{q}}{∥F_{s}∥\cdot ∥F_{q}∥},$$ (16)

$$P_{\text{aux}}=C\left(ℛ\left(D_{\text{cos}}\right),P_{e}\right),$$ (17)

where  ⋅  denotes the dot product, and $P_{aux}$ represents effective defect perception information, serving as an auxiliary part in the feature space to alleviate the distortion intra-class difference.

### Information fusion module

The local-view information $P_{main}$ and global-view information $P_{aux}$ are integrated as a guidance information via IFM for the surveillance of query image segmentation. We employ two residual Connections (Res) and a classification head (Cls) as the IFM to feed the $P_{main}$ and $P_{aux}$ to predict the mask ${\tilde{M}}_{q}$ for image $I_{q}$:


$$\begin{array}{c} {\tilde{M}}_{q}=F\left(R\left(C\left(P_{main},P_{aux}\right)\right)\right), \end{array}$$ (18)

where *C* ( ⋅ )  concatenates $P_{main}$ and $P_{aux}$, *R* ( ⋅ )  reshapes the input to the same size as $M_{q}$, and *F* ( ⋅ )  indicates the Res and Cls. For the sake of simplicity, it is described by Algorithm 1.

## Experiments

### Experimental settings

*Datasets*: For assessment of our model, experiments are accomplished on two metal surface defect FSS datasets, Surface Defect-$4^{i}$ [13] and FSSD-12 [38]. The total 12 metal surface defect classes in the Surface Defect-$4^{i}$ are divided homogenously into 3 folds *i* ∈ 0 , 1 , 2, with every fold encompassing 4 classes. Like Surface Defect-$4^{i}$, 12 strip steel surface defect classes in FSSD-12 are partitioned into 3 folds, each containing 4 classes.

*Evaluation Metrics:* Consistent with conventional FSS approaches, the mean and foreground-background intersections-over-unions (mIoU and FB-IoU) are used as our evaluation metrics. FB-IoU directly computes the average of foreground and background IoUs without considering object classes, while mIoU computes the average of IoUs for the entire classes in a fold. Furthermore, mIoU is used to describe the overall performance of per algorithm.

*Implementation Details:* ResNet-50 [39] and VGG-16 [40], which have been pretrained on Surface Defect-$4^{i}$ and FSSD-12 datasets, are used as our backbone networks and PyTorch 1 . 12 . 1 is used for training 200 epochs via the SGD optimizer. For uniformity and consistency, we standardize the input image to a 200 × 200 size. In the process of training, the training and test batch sizes are set to 2 and 1, respectively, and Nvidia GeForce RTX 3090 (20G) GPUs are utilized for the entire experiments.

*Baseline:* For baseline establishment, the three utilized modules are removed from LDMP-RENet, leaving two Res and one Cls to guide downstream tasks. To achieve final segmentation, the baseline leverages the backbone-extracted features, which are concatenated as input for downstream tasks. Moreover, the loss computation in this method resembles that in LDMP-RENet.

**Algorithm 1: L**ocal **D**escriptor-based **M**ulti-**Prototype** **R**easoning and **E**xcitation **Net**work (LDMP-RENet)

**Input:** A training dataset $D_{train}$

**Output:** Trained parameters

**1** **for** *each episode ${\left\{\left(I_{s}^{k},M_{s,c}^{k},I_{q},M_{q,c}\right)\right\}}_{c\in C_{episode}}^{k=1,\ldots ,K}\in D_{train}$* **do**

**2**    Extract $F_{q}$ and $F_{s}$ from backbone and extend to *K*-shot by Eq (1);

**3**    Represent $F_{s}$ and $F_{q}$ as local descriptors $X_{s}$ and $X_{q}$;

   // Multi-Prototype Reasoning:

**4**    Embed to graph space by Eqs (6)–(7);

**5**    Obtain node and channel level relevance $G_{sq}^{v}$ and $G_{sq}^{d}$ by Eqs (8)–(9);

**6**    Concatenate $G_{sq}^{v}$ and $G_{sq}^{d}$ to get $G_{sq}$ by Eq (10);

**7**    Reasoning $G_{sq}$ through GCN to get *G* by Eqs (2)–(4);

**8**    Reflect back into feature space, and then get $P_{main}$ by Eq (11);

   // Multi-Protorype Excitation:

**9**    Get foreground information $X_{proj}$ by Eq (12);

**10**    Generate foreground multi-prototype $P_{proj}$ by Eq (13);

**11**    Obtain multi-prototype $P_{e}$ by Eqs (14)–(15);

**12**    Calculate global edge information $D_{cos}$ by Eq (16);

**13**    Concatenate $P_{e}$ and $D_{cos}$ to get multi-prototype $P_{aux}$ by Eq (17);

   // Information Fusion Module:

**14**    Fuse $P_{main}$ and $P_{aux}$ by Eq (18) to get ${\tilde{M}}_{q}$;

**15**    Compute binary cross entropy Loss $L_{BCE}$ by Eq (5);

**16** **end**

### Comparison with state-of-the-arts

*Surface Defect-$4^{i}$:* Table 1 presents the performance comparison of our model with a few representative models on Surface Defect-$4^{i}$. As is clear, (1) LDMP-RENet delivers unprecedented performance in both the 1-shot and 5-shot settings. In particular, for the VGG-16 backbone, it outperforms TGRNet and CPANet, which previously had the most advanced surface defect detection results for metal generic and strip steel, by 13 . 07*%* (1-shot) and 15 . 79*%* (1-shot). The primary reason is the contribution made by MPR and MPE towards reducing intra-class differences in metal surface defects. (2) LDMP-RENet exhibits a huge performance difference, gaining 12 . 41*%* and 10 . 83*%* advantages over its nearest competitor in VGG-16. The substantial disparity exhibited on Surface Defect-$4^{i}$ underscores the state-of-the-art level and effectiveness of our LDMP-RENet. (3) Compared with VGG-16, the improvement of our network under ResNet-50 is limited, with a gap of 3 . 56*%* (1-shot) and 3 . 54*%* (5-shot) respectively from the second place. The reason is that as the network depth increases, it is diﬃcult to gather high-level defect semantic features from the GEI in the mid-level. Unlike mid-level features, the mode-related high-level features represent the overall semantic information of metal surface defect categories, such as patches and spots on steel surfaces and scratches on aluminum surfaces. (4) LDMP-RENet performs prominently better than the baseline model. For instance, in the VGG-16 backbone, LDMP-RENet and baseline achieve 39 . 50*%* and 23 . 08*%*, respectively. This underscores the stability of performance facilitated by MPR and MPE.

**Table 1:** Compare with state-of-the-art metal surface defect FSS and amateur networks on Surface Defect-$4^{i}$ in mIoU and FB-IoU under 1-shot and 5-shot. The best and second best results are highlighted with bold and underline

Methods	Backbone	1-shot					5-shot				
		Fold-0	Fold-1	Fold-2	mIoU	FBIoU	Fold-0	Fold-1	Fold-2	mIoU	FBIoU
DCPNet [41]	VGG-16	28.68	27.45	24.08	26.74	52.22	22.91	27.81	25.59	25.44	51.96
HDMNet [42]		31.10	28.91	21.26	27.09	52.76	42.33	28.00	25.97	32.10	51.45
PFENet [43]		23.28	19.45	20.48	21.07	51.14	27.94	21.67	25.24	24.95	53.99
TGRNet(1-normal) [13]		29.78	25.15	24.36	26.43	51.50	37.42	24.66	26.52	29.53	53.27
CPANet [38]		22.03	25.05	24.07	23.72	51.35	30.11	25.95	19.26	25.11	52.21
*Baseline*		*28.33*	*24.54*	*16.38*	*23.08*	*49.12*	*32.11*	*24.87*	*19.92*	*25.63*	*51.67*
**LDMP-RENet (Ours)**		**51.61**	**39.07**	**27.83**	**39.50**	**58.15**	**56.74**	**41.75**	**30.30**	**42.93**	**59.83**
DCPNet [41]	ResNet-50	27.19	31.96	24.68	27.94	51.67	42.78	39.35	32.21	38.11	58.77
HDMNet [42]		35.58	**40.79**	27.50	34.62	56.01	38.62	41.11	**32.61**	37.45	56.19
PFENet [43]		29.45	24.90	16.21	23.52	54.06	33.98	30.07	22.78	28.94	56.92
TGRNet(1-normal) [13]		35.46	32.37	24.75	30.86	53.62	41.61	28.66	27.87	32.71	53.00
CPANet [38]		32.52	29.65	24.66	28.94	51.94	39.36	37.84	27.82	35.01	57.73
*Baseline*		*34.84*	*24.17*	*19.63*	*26.21*	*49.42*	*34.55*	*24.53*	*23.98*	*27.69*	*50.66*
**LDMP-RENet (Ours)**		**46.10**	39.60	**28.85**	**38.18**	**57.16**	**50.90**	**42.41**	31.65	**41.65**	**59.17**

*FSSD*-12: FSSD-12 is a dataset that only contains strip steel surface defect. Relevant performance comparisons are detailed in Table 2. In general, LDMP-RENet outperforms all the existing models in both 1-shot and 5-shot settings. It leads TGRNet and CPANet by 1 . 33*%* and 0 . 90*%* on VGG-16 in the 1-shot setting. Compared to the performance on Surface Defect-4^i^, our network shows little improvement on FSSD-12. There are two possible reasons: (1) From the baseline performance of the two datasets, there is a 19 . 05*%* gap between the two under VGG-16, showing that Surface Defect-$4^{i}$ is more diﬃcult to process than FSSD-12. This forces the relevant network to optimize the common problems existing in general metal surface defects, i.e., the intra-class difference problem. (2) As this problem becomes more severe, the related networks are not optimized for it, causing lower accuracy. The proposed MPR and MPE address this by using local and global view information to mitigate semantic and distortion intra-class differences, respectively, ultimately resolving the intra-class difference issue for metal surface defects, achieving the most advanced performance.

**Table 2:** Compare with state-of-the-art metal surface defect FSS and amateur networks on FSSD-12 in mIoU and FB-IoU under 1-shot and 5-shot. The best and second best results are highlighted with bold and underline

Methods	Backbone	1-shot					5-shot				
		Fold-0	Fold-1	Fold-2	mIoU	FBIoU	Fold-0	Fold-1	Fold-2	mIoU	FBIoU
DCPNet [41]	VGG-16	53.30	44.52	40.98	46.27	66.01	50.07	48.69	43.07	47.28	67.28
HDMNet [42]		50.12	49.05	45.51	48.23	66.57	48.26	49.60	46.11	47.99	66.69
PFENet [43]		43.65	37.89	36.12	39.22	67.72	44.86	40.66	36.57	40.70	68.92
TGRNet(0-normal) [13]		63.74	51.68	49.95	55.12	73.36	66.16	**60.24**	51.07	59.16	74.48
CPANet [38]		50.90	47.39	**53.38**	50.56	69.26	50.15	37.41	43.39	43.65	64.73
*Baseline*		*48.40*	*38.04*	*39.94*	*42.13*	*63.94*	*48.85*	*43.98*	*41.84*	*44.89*	*65.24*
**LDMP-RENet (Ours)**		**67.60**	**52.00**	49.74	**56.45**	**74.75**	**68.38**	59.01	**52.78**	**60.06**	**75.23**
DCPNet [41]	ResNet-50	59.65	63.13	51.84	58.21	74.79	61.68	61.80	52.71	58.73	74.10
HDMNet [42]		60.50	**65.46**	51.42	59.13	74.33	63.03	68.22	53.74	61.66	77.33
PFENet [43]		49.00	47.87	41.78	46.22	73.76	50.11	50.98	42.34	47.81	74.77
TGRNet(0-normal) [13]		61.09	63.24	51.28	58.54	75.20	61.59	65.81	56.27	61.22	76.74
CPANet [38]		54.40	52.59	48.39	51.79	65.15	56.73	55.06	51.92	54.57	71.19
*Baseline*		*53.95*	*43.53*	*42.92*	*46.80*	*64.66*	*55.84*	*50.24*	*43.57*	*49.88*	*67.74*
**LDMP-RENet (Ours)**		**62.60**	62.63	**52.95**	**59.39**	**75.66**	**64.52**	**64.21**	**56.35**	**61.69**	**77.51**

*Qualitative Results:* For a more profound analysis and comprehension of our model, we employ a twofold strategy for visual examination: an internal component comparison to dissect the model’s mechanics, and an external benchmarking against leading-edge techniques to contextualize its performance.

**Fig 5:**
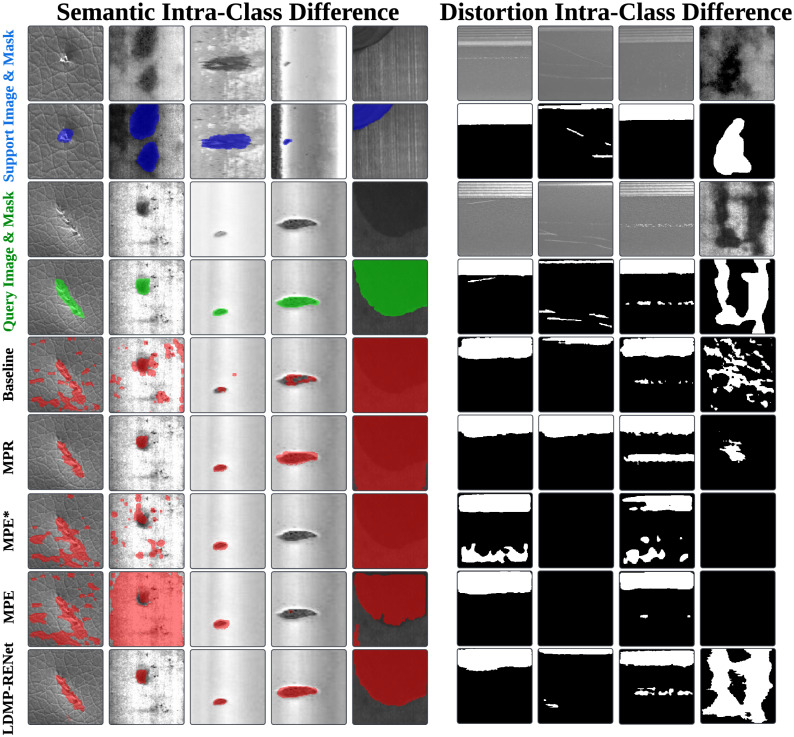
Qualitative results of baseline and components of our LDMP-RENet on Surface Defect-$4^{i}$. Contrary to MPE, MPE* lacks the incorporation of global edge information $D_{cos}$

**Fig 6:**
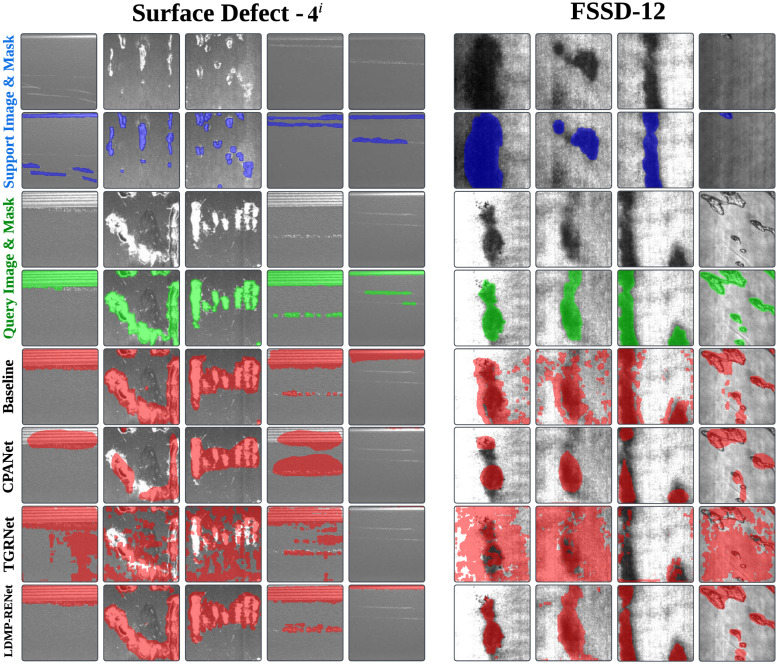
Qualitative outcomes for the baseline, CPANet,TGRNet and our LDMP-RENet in 1-shot setting. The left panel is from Surface Defect-$4^{i}$, and the right one is from FSSD-12. Each row from top to bottom represents the support images with ground-truth (GT) masks (blue), query images with GT masks (green), CPANet results (red), TGRNet results (red), and our results (red), respectively. The 1st to 3rd and 6th to 9th columns correspond to semantic intra-class difference, whereas the 4th and 5th columns illustrate the distortion intra-class difference.

1) Fig 5 presents the visualization of performance for the submodules within LDMP-RENet. As shown in Fig 5, the baseline (5th row) fails to accurately capture both local and global features. The MPR module (6th row) adeptly captures local-view features, although it may overlook global context. Conversely, comparing with MPE* (7th row), the MPE module (8th row) excels at gaining global-view information. Leveraging the complementary strengths of MPE and MPR, LDMP-RENet (the last row) achieves enhanced segmentation performance. On the industrial pipeline, samples exhibit extreme intra-class variations, where the wear types in the support and query sets are consistent, yet the distribution of the foreground (local) and background (global) are starkly different. LDMP-RENet, integrating MPR technology for capturing local features and MPE technology for focusing on global information, facilitates its adaptation to defect detection tasks characterized by extreme intra-class differences (3rd to 5th columns in Fig 5), thereby yielding more precise segmentation outcomes.

2) As displayed in Fig 6, LDMP-RENet is subjected to effectiveness assessment through the segmentation results visualization. In this context, columns 1, 4, and 5 depict defects of aluminium surfaces, whereas the remaining columns illustrate defects of steel surfaces. The segmentation eﬃciency of our LDMP-RENet is superior to TGRNet and CPANet across Surface Defect-$4^{i}$ and FSSD-12. (1) Although TGRNet and CPANet struggle with managing intra-class differences within their respective datasets, our algorithm precisely segments the target across both datasets. (2) CPANet is characterized by an attention mechanism and lacks the concatenation of local cues, while LDMP-RENet enables local-view feature excavation for varying fine-grained classes, therefore tackling the semantic intra-class difference (1st to 3rd and 6th to 9th columns). (3) TGRNet explores the direct connection of support-query pairs within the overall prototype and lacks global-local interaction, while LDMP-RENet can provide global-view features through multi-prototype excitation (4th to 5th columns), addressing the distortion intra-class difference.

*Industrial Performance:* Highlighting cost eﬃciency under industrial scenarios, we compare the FLOPs along with the parameters in Table 3. Clearly, our model displays lower FLOPs and fewer parameters, which is conducive to faster and more precise completion of the metal surface defect segmentation.

**Table 3:** Comparison of model performance on Surface Defect-$4^{i}$. “FLOPs" indicates the computational overhead. “#Params." indicates the number of learnable parameters

Methods	mIoU	FB-IoU	FLOPs	#Params.
TGRNet [13]	30.86	53.62	83.69G	9.38M
CPANet [38]	28.94	51.94	162.23G	11.98M
**LDMP-RENet (Ours)**	**38.33**	**58.77**	**69.85G**	**8.28M**

### Robustness analysis

**Fig 7:**
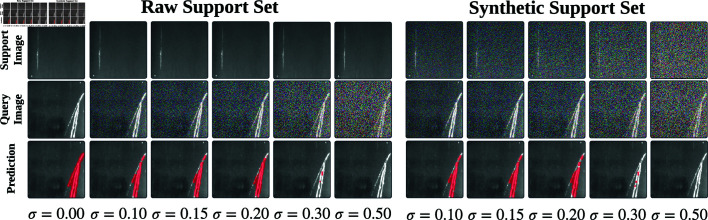
Qualitative analysis of LDMP-RENet with different noise on Surface Defect-$4^{i}$.

In high-speed industrial pipelines, samples are subjected to a myriad of complex and variable external disturbances, including variations in lighting and humidity, which pose a rigorous challenge to the robustness of defect detection algorithms. LDMP-RENet performs robustness experiments under two distinct scenarios: where either the support-query pairs (synthetic support set) or solely the query images (raw support set) are affected by noise. As illustrated in Fig 7, the variance *σ* of the random distribution is varied across values of 0 . 10 , 0 . 15 , 0 . 20 , 0 . 30 and 0 . 50 to assess the model’s performance under both the raw and synthetic support sets. The findings indicate the following: (1) When *σ* < 0 . 30, the model is able to effectively leverage defect features from both the raw and synthetic support sets to guide accurate segmentation of the query image (2nd to 4th columns on the left and 1st to 3rd columns on the right). (2) When *σ* ≥ 0 . 30, the increased noise or uncertainty in the input data disrupts the whole segmentation performance of LDMP-RENet.

### Ablation study

For the effectiveness verification of three proposed modules, i.e., MPR, MPE* and MPE, where MPE* lacks the incorporation of global edge information ***D***_cos_, extensive ablation experiments are carried out on Surface Defect-4^i^ dataset in the 1-shot setting with the utilization of VGG-16 backbone in this section.

*Components Analysis:* The effects of various components on model performance are detailed in Table 4. Application of two proposed components enhances the baseline by 16 . 42*%*. In the first row, MPR mines the local-view features and improves the baseline by 13 . 61*%*. In the second row, MPE* lowered the baseline 2 . 64*%*. This phenomenon arises due to the activation of background texture information, which the model erroneously interprets as pertinent information. By using Dcos to collect the defect information from activated multi-prototype, the baseline yields an 8 . 29*%* performance gain. With the combination of MPR and MPE*, the baseline is improved by 13 . 71*%*. This indicates that the improvement of MPR over the baseline is not compromised by the negative effects of MPE*. In addition, it demonstrates the stability of the local descriptor-based semantic information extracted by MPR. After the combination of MPR with MPE, IFM is employed to integrate local-view and global-view features, thereby allowing the model to mitigate intra-class difference issue, and to effectively outperform the baseline by 16 . 42*%*.

**Table 4:** Ablation studies on each component on the Surface Defect-4^i^. Contrary to MPE, MPE* lacks the incorporation of global edge information $D_{cos}$

MPR	MPE*	MPE	1-shot			
			Fold-0	Fold-1	Fold-2	mIoU
*✓*			50.80	35.08	24.19	36.69_↑13.61_
	*✓*		28.49	25.25	23.42	25.72_↓2.64_
		*✓*	39.25	27.08	27.80	31.38_↑8.29_
*✓*	*✓*		50.88	35.15	24.57	36.87_↑13.78_
*✓*		*✓*	**51.61**	**39.07**	**27.83**	**39.50_↑16.42_**
Baseline			*28.33*	*24.54*	*16.38*	*23.08*

Additionally, we performed ablation studies targeting the adaptability of MPR. As demonstrated in Table 5, MPR imparts adaptive capabilities to each node level prototype, resulting in a 2 . 43*%* improvement in overall performance compared to conventional graph reasoning. This is attributed to the introduction of adaptive capability, which enables the model to adapt the diversity in the appearance of metal surface defects, thereby facilitating the effective extraction of semantic features to address intra-class differences.

**Table 5:** Ablation studies on GCN and MPR on the Surface Defect-$4^{i}$. GCN indicates the conventional graph reasoning

GCN	MPR	1-shot			
		Fold-0	Fold-1	Fold-2	mIoU
*✓*		50.12	34.55	26.56	37.08
	*✓*	**51.61**	**39.07**	**27.83**	**39.50**

*Ablation Experiment of the Backbone:* To validate the superiority of our backbone, we subject various ResNet and VGG networks to ablation experiments as detailed in Table 6. Obviously, ResNet-50 and VGG-16 have better performance than others. From our perspective, the depth of ResNet and VGG layers correlates with an increased risk of overfitting. By contrast, with a reduced number of layers (such as ResNet-18 and ResNet-34), the models exhibit diﬃculties in capturing complex features. Consequently, ResNet-50 and VGG-16 achieve a more optimal balance and are thus better suited for fulfilling task requirements.

**Table 6:** 1-shot mIoU and FB-IoU of ablation study for Resnet and VGG.

Backbone	1-shot			
	Fold-0	Fold-1	Fold-2	mIoU
VGG16	51.61	39.07	**27.83**	**39.50**
VGG19	**52.01**	**39.92**	16.43	36.12
ResNet18	41.64	35.77	26.38	34.60
ResNet34	44.02	37.77	23.99	35.26
ResNet50	**46.10**	**39.60**	**28.85**	**38.18**
ResNet101	45.88	38.56	25.87	36.77
ResNet152	42.72	36.11	24.72	34.52

**Fig 8:**
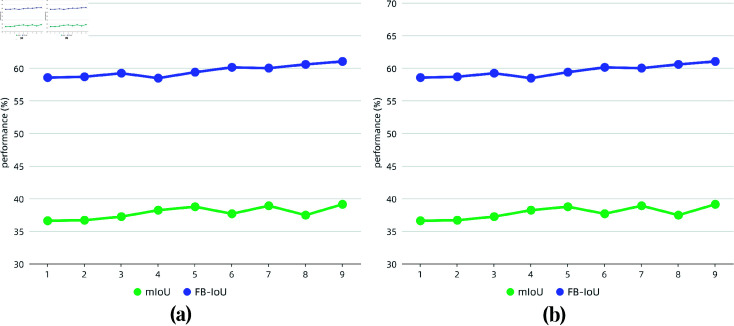
Ablation experiment of *K*-shot on Surface Defect-$4^{i}$. (a) and (b) denote the *K*-shot performance of VGG-16 and ResNet-50 respectively.

*Ablation Experiment of *K*-shot:* For the number of support images *K*, the *K* masked support features are averaged to input the network. As described in the left image of Fig 8, the network performance is about to improve with the increasing *K* value. This is similar to the prior research findings on few-shot semantic segmentation.

*Computational Costs:* Table 7 illustrates that the LDMP-RENet, which integrates both the MPR and MPE modules, exhibits a minimal increase in FLOPs and #Params. relative to other individual components. This indicates that the combination of components does not incur a significant computational overhead, making LDMP-RENet suitable for industrial applications.

**Table 7:** Computational costs with LDMP-RENet on Surface Defect-$4^{i}$.

MPR	MPE*	MPE	FLOPs	#Params.
*✓*			68.77G	8.26M
	*✓*		68.73G	8.06M
		*✓*	68.83G	8.06M
*✓*	*✓*		69.75G	8.28M
*✓*		*✓*	69.85G	8.28M
Baseline			47.06G	8.12M

### Application scenario

**Fig 9:**
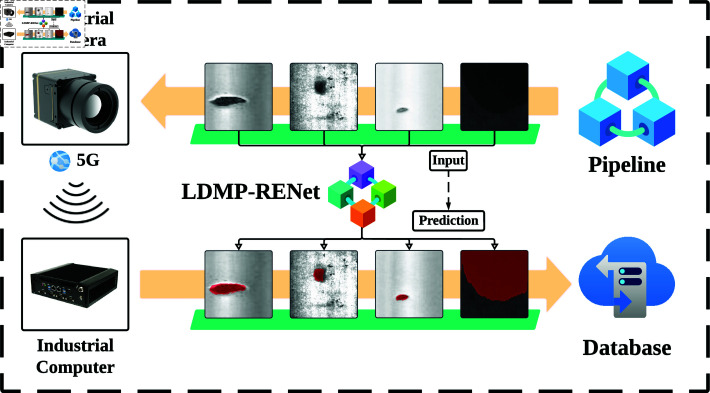
Pipeline working mode based on LDMP-RENet.

LDMP-RENet is designed to provide a more eﬃcient solution for the detection task of metal surface defects in pipeline. It utilizes a local descriptor-based few-shot segmentation model and high-speed industrial cameras to enhance the accuracy and speed of metal surface defect detection. The traditional pipeline systems for metal surface defect detection are constrained by a limited number of signal transmission lines, which inevitably prolong the overall detection time and reduce eﬃciency [21]. To address this issue, we propose an innovative solution by integrating LDMP-RENet with 5G wireless transmission. As shown in Fig 9, the industrial camera, with its high exposure and fast shutter capabilities, quickly captures a large number of metal surface defect samples on the pipeline. These samples are then transmitted via 5G signals to the industrial computer. There, LDMP-RENet is deployed to achieve high-precision defect segmentation, with the results being meticulously stored in the database.

## Conclusion

To conclude, in this study, an innovative FSS network is introduced, which leverages local descriptors, multi-prototype reasoning, and activation to diminish intra-class difference in metal surface defects. MPR exploits graph reasoning to discern interrelations among local descriptors within graph space, capturing local-view features to alleviate semantic intra-class difference. Conversely, MPE harnesses global edge information in feature space to navigate activated descriptors, targeting the rectification of distortion intra-class difference through global-view features. The IFM amalgamates data from both graph and feature spaces, achieving more precise segmentation. Extensive testing confirms that LDMP-RENet consistently delivers cutting-edge performance across various configurations. In comparison with the time series anomaly detection algorithm used in [44,45], LDMP-RENet is limited to the recognition of static images, which may not perform well on the random elastic deformation of metals caused by objective factors such as speed fluctuations and vibrations of continuous rolling equipment. Therefore, time series analysis can be used to solve such problems.
